# Investigations Into the Human Metabolism of Trestolone (7α‐Methyl‐19‐Nortestosterone)

**DOI:** 10.1002/dta.70018

**Published:** 2025-12-17

**Authors:** Thomas Piper, Gregor Fusshöller, Mario Thevis

**Affiliations:** ^1^ Center for Preventive Doping Research—Institute of Biochemistry German Sport University Cologne Köln Germany; ^2^ European Monitoring Center for Emerging Doping Agents (EuMoCEDA) Cologne/Bonn Köln Germany

**Keywords:** doping controls, high‐resolution mass spectrometry, human metabolism, hydrogen isotope ratios, isotope ratio mass spectrometry

## Abstract

Already in the 1960s, the anabolic properties of Trestolone (7α‐methyl‐19‐nortestosterone, MENT) were investigated in the context of cancer research, and MENT was found to be 10 times more potent regarding its anabolic properties compared to testosterone. The human metabolism of MENT was only investigated once in an antidoping context, and three urinary metabolites were identified, corroborating earlier findings from in vitro and animal experiments. Based on these metabolites, no doping control sample was reported to contain MENT or its metabolites in the last two decades albeit MENT is readily available via online distributors. One reason for the lack of adverse analytical findings in doping controls could be analytical challenges originating from the chromatographic properties of MENT and its urinary metabolites. Therefore, the human metabolism of MENT was reinvestigated employing an excretion study with deuterated MENT and metabolite detection based on hydrogen isotope ratio mass spectrometry in combination with high accuracy/high resolution mass spectrometry. Considering unconjugated, glucuronidated, and sulfated metabolites, 50 potential candidates were detected. In order to identify those metabolites suitable for sports drug testing, three volunteers administered a single oral dose of nondeuterated MENT, and all postadministration samples were investigated using triple quadrupole mass spectrometry‐based determinations routinely employed in doping controls. From the 50 metabolites detected, two showed promising results with respect to their detection windows and suitability under routine measurement conditions. The specificity of the novel metabolites was ensured by the reanalysis of 200 routine doping control samples demonstrating the absence of potential coeluting compounds.

## Graphical abstract

As the known metabolites of 7α‐methyl‐19‐nortestosterone (MENT), M1 and M2, suffer from chromatographic issues under routine doping control conditions, the human metabolism of MENT was reinvestigated. From the numerous new metabolites detected, especially, M5 and M10 were identified as the most promising candidates to be implemented into routine procedures. Both enable the detection of MENT misuse following a single oral administration for up to 4 days.

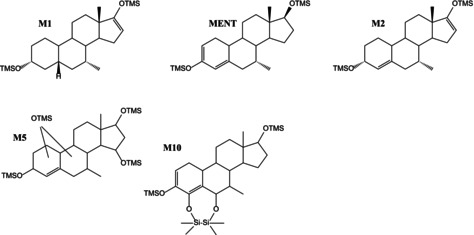

## 1 Introduction

**FIGURE 1:**
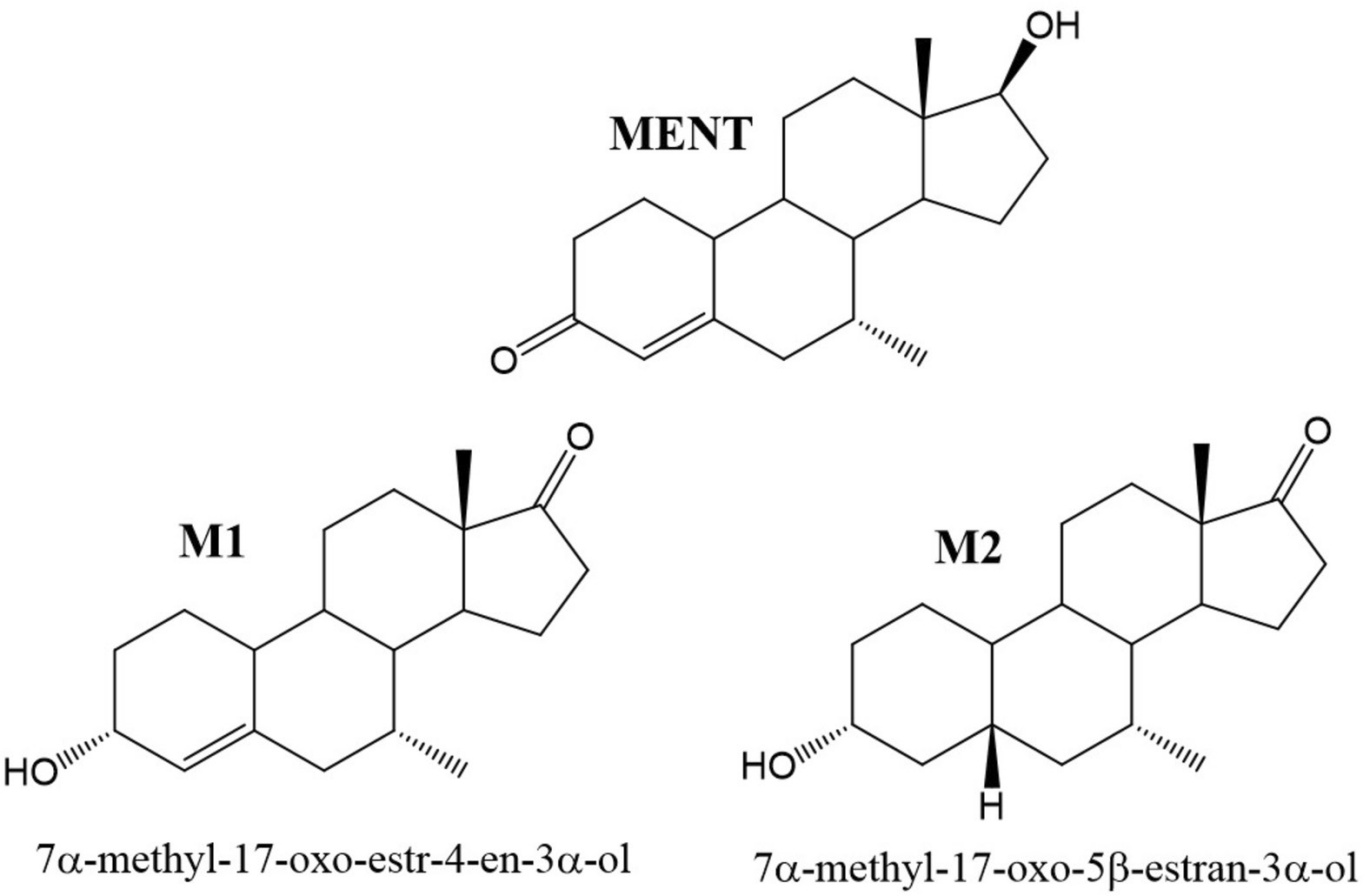
Chemical structures of MENT (7α‐methyl‐19‐nortestosterone) and its metabolites described to be produced in vivo.

The anabolic properties of 7α‐methyl‐19‐nortestosterone (MENT, Figure 1) have been investigated in the context of cancer research as early as the 1960s [1, 2]. MENT was found to be 10 times more potent regarding its anabolic properties compared to testosterone, and due to the 7α‐methyl moiety, the endogenous metabolism towards 5α‐reduced metabolites was minimized. This, in combination with the relatively fast metabolic serum clearance rate induced by the low binding affinity to sex hormone‐binding globulin (SHBG), made MENT also a promising candidate for hormonal male contraception and hormonal replacement therapy [3, 4]. Nevertheless, MENT never received full approval for medical use.

Regarding sports drug testing, the human metabolism of MENT was only investigated once, and three urinary metabolites were identified, corroborating earlier findings from in vitro and animal experiments [5, 6]. All urinary metabolites described and identified so far are summarized in Figure 1, including the parent compound MENT, 7α‐methyl‐17‐oxo‐estr‐4‐en‐3α‐ol (M1), and 7α‐methyl‐17‐oxo‐5β‐estran‐3α‐ol (M2). After oral administration, MENT and its metabolites were detectable for up to 14 h.

Albeit MENT can easily be purchased via internet‐based providers marketed as a nutritional supplement, and despite its constant availability, MENT did never result in an adverse analytical finding according to the Testing Figure Reports issued by WADA since 2003. This may be due to chromatographic issues with the reported metabolites or the relatively short detection window.

**FIGURE 2:**
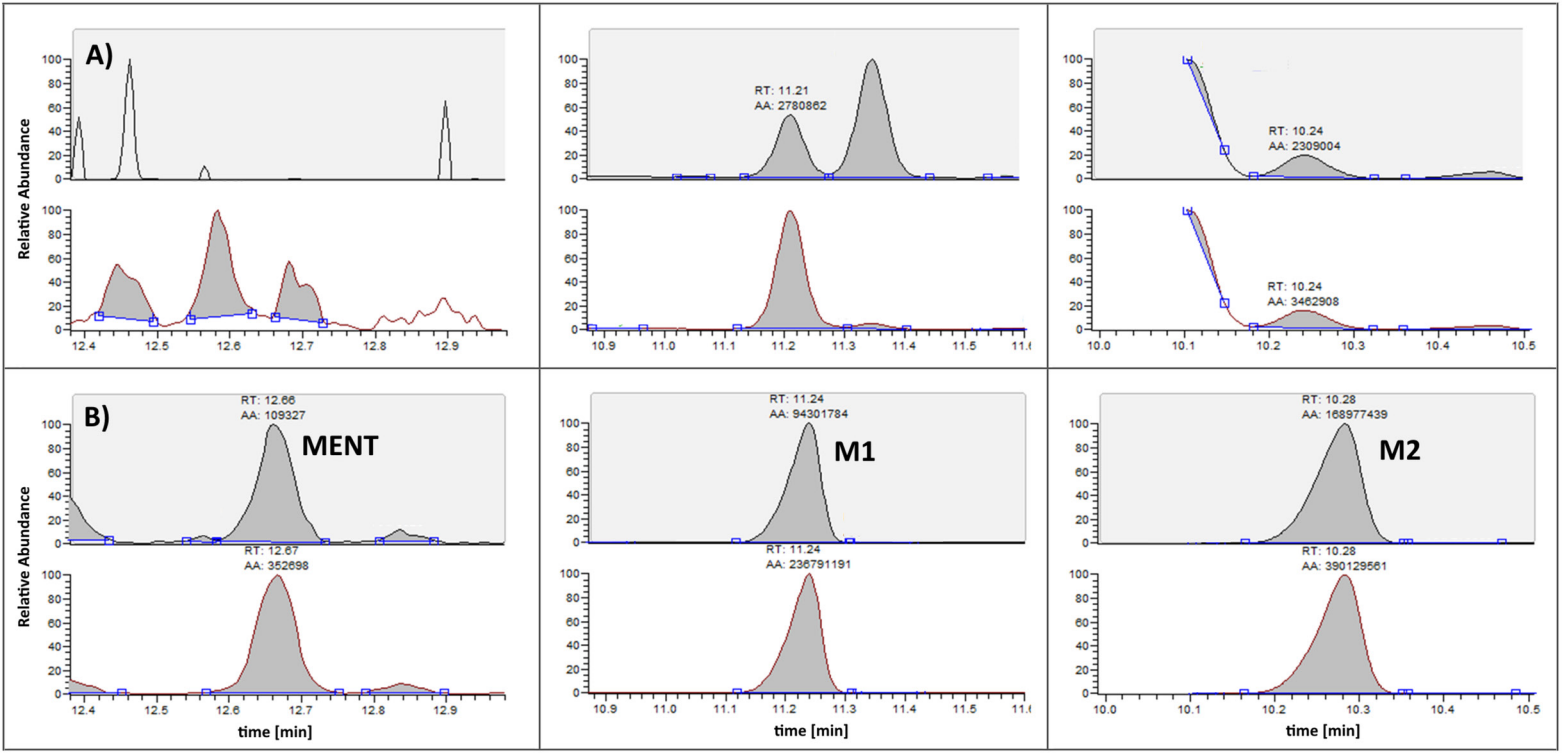
GC/MS‐chromatograms obtained from the blank urine sample before the administration of 30 mg of MENT‐Ac (A) and 6 h after (B). Further information in the text.

The main problem with both metabolites is their structural similarity to many endogenous C19‐steroids resulting in comparable retention times and identical ion transitions produced by many other endogenous steroids present in the test matrix (i.e., urine). This is exemplarily demonstrated in Figure 2. All investigated truly negative urine samples show abundant signals at the expected retention times of M1 and M2; only MENT itself is not as strongly affected in some samples. As no urinary concentration threshold for MENT or its metabolites was established so far, differentiating between a MENT administration and an endogenous coelution remains challenging.

To overcome this limitation and to improve its detectability, the human metabolism of MENT was reinvestigated employing deuterated MENT acetate (MENT‐Ac) and hydrogen isotope ratio mass spectrometry (IRMS) in a first step, facilitating the identification of a comprehensive set of urinary metabolites. This approach has already been successfully employed in sports drug testing several times in the past [7, 8, 9, 10]. Confirmation of potentially deuterated compounds and a basic structural elucidation of observed deuterated metabolites was accomplished employing high resolution/high accuracy mass spectrometry (HRMS).

As some metabolites of MENT were expected to coelute with endogenous steroidal analytes as described above, the suitability of newly identified potential markers for sports drug testing purposes was to be assessed. This was ensured by investigations into additional administrations trials employing nondeuterated MENT‐Ac administered to three volunteers in order to partially cover the expected biological variation and identify those metabolites suitable for implementation into routine screening procedures.

## 2 Experimental

### 2.1 Chemicals and Steroids

Certified steroid reference material for MENT, M1 and M2 was purchased at the National Measurement Institute (Canberra, Australia). Steroid reference material MENT was also available from Toronto Research Chemicals via LGC (Luckenwalde, Germany) and used for the deuteration experiments. Two nutritional supplements containing MENT‐Ac were purchased via the world wide web. The internal standard 17α‐methyltestosterone (MeT), pregnanediol (PD), 16‐androstenol (EN), deuterium oxide (D_2_O, 99.9 atom %), methanol‐D (MeOD, 99.5%), deuterium chloride (DCl, 20% in D_2_O), sodium deuteroxide (NaOD, 40% in D_2_O), and sulfuric acid were from Sigma Aldrich (Steinheim, Germany). Methanol (MeOH), *tert*.‐butyl methyl ether (TBME), acetonitrile (ACN), ethyl acetate (EtOAc), ethanethiol, and ammonium iodide (NH_4_I) were purchased from Merck (Darmstadt, Germany). N‐methyl‐N‐trimethylsilyltrifluoroacetamide (MSTFA) was purchased from Chemische Fabrik Karl Bucher (Waldstetten, Germany). All solvents and reagents were of analytical grade. The β‐glucuronidase from *Escherichia coli* employed for enzymatic hydrolysis was from Roche Diagnostics GmbH (Mannheim, Germany). Solid phase extraction (SPE) cartridges Chromabond C18 (500 mg) were obtained from Macherey & Nagel (Düren, Germany). Helium (purity 5.0 and 4.6), nitrogen (purity 5.0), and the H_2_ tank gas (purity 3.9) were from Linde (Pullach, Germany). The tank gas was calibrated against n‐alkanes comprised in the secondary reference material A6 provided by A. Schimmelmann at the Indiana University (Bloomington, IN, United States).

### 2.2 Deuteration of MENT

**FIGURE 3:**
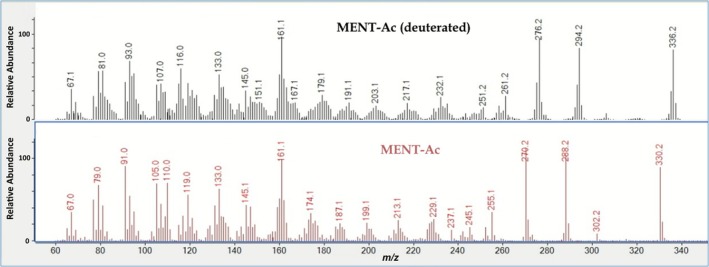
Low‐resolution mass spectra obtained on sixfold deuterated MENT‐Ac and nondeuterated MENT‐Ac.

The deuteration of MENT was based on established protocols [10, 11]. Fifty milligrams of MENT were dissolved in MeOD (5 mL), acidified with 80 μL of DCl, and heated to 60°C overnight. After cooling to room temperature, the mixture was neutralized by adding 50 μL of NaOD and evaporated to dryness. The dry residue was reconstituted in 5 mL of D_2_O, vortexed, and extracted twice with 5‐mL TBME. The organic layers were combined and evaporated to dryness, yielding 44 mg of deuterated MENT. Acetylation was performed by adding 0.25 mL of acetic anhydride and pyridine, each, and heating to 70°C for 1 h. After cooling to room temperature, the sample was evaporated to dryness. A low‐resolution mass spectrum of both, the sixfold deuterated, and native MENT‐Ac is shown in Figure 3. Based on the chemical structure, deuteration was expected to take place in the A and B rings of the molecule. Minute amounts of nonacetylated MENT were present in the final product representing less than 2% of the acetylated MENT and were considered not to have any detrimental effect on the planned excretion study.

### 2.3 Administration Trials

One male volunteer (49 years, 180 cm, 84 kg) administered 30 mg of deuterated MENT‐Ac dissolved in 5 mL of a mixture of ethanol and water (20%, v/v). Three blank urine samples were collected at different time points the day before the administration took place. During the first 48 h after ingestion, all urine sample were collected; then, a morning and an evening sample was collected for the next 4 days. All samples were stored frozen until analysis.

Nondeuterated MENT‐Ac (30 mg) was administered dissolved in the same mixture of ethanol and water as described by three male volunteers (sport students at the German Sport University Cologne) after collection of one blank urine sample followed by all urine samples for the first 48 h and a morning and an evening urine samples until 7 days (162 h) after the administration. All samples were stored frozen until analysis.

All volunteers were informed and gave written consent, and the local ethics committee of the German Sport University Cologne, Germany, approved the study (Approval # 052/2023).

### 2.4 Athlete Population for Specificity Assessment

In order to assess the specificity of novel MENT metabolites, 200 athlete urine samples were investigated to ensure the absence of potentially coeluting compounds and to optimize the chosen multiple reaction monitoring (MRM) ion transitions by excluding those impaired by the biological matrix. The samples were chosen arbitrarily and represented 200 specimens with consecutive internal laboratory numbers. This resulted in *n* = 113 male and *n* = 87 female samples of which 33.5% were collected in competition representing 27 different sport disciplines. All samples were prepared according to the routine screening procedure and reinjected for this research project [12, 13].

### 2.5 Sample Preparation Protocol for Metabolite Identification

To investigate unconjugated, glucuronidated, and sulfated urinary metabolites separately, the sample preparation protocol enabled the fractionation of these Phase II metabolites as demonstrated previously [7, 8, 9, 10]. In brief, 20 mL of urine was applied to an equilibrated SPE (2 mL of MeOH followed by 2 mL of water), washed with 2 mL of water, and eluted with 3 mL of MeOH. Samples were evaporated to dryness and reconstituted with 2 mL of phosphate buffer (pH = 7). Unconjugated steroids were extracted by adding 5 mL of TBME, shaking, centrifugation, and transfer of the organic layer to a new test tube followed by evaporation to dryness. Glucuronidated steroids still present in the aqueous phase were deconjugated by adding 100 μL of β‐glucuronidase and incubating for 1 h at 50°C. After cooling to room temperature, 1 mL of carbonate buffer (pH = 10) was added. Samples were vortexed, and formerly glucuronidated steroids were extracted by adding 5 mL of TBME, shaking, centrifugation, and transfer of the organic layer to a new test tube followed by evaporation to dryness. The aqueous phase still containing the sulfated steroids was acidified by adding 100–200 μL of glacial acetic acid before application to conditioned SPEs. Samples were washed with 2 mL of water and eluted with 3 mL of MeOH before taking to dryness. In order to deconjugate the sulfate moiety, 2.5 mL of a mixture of MeOH/EtOAc (70/30, v/v) and 1 mL of a mixture of ethyl acetate/sulfuric acid (100 mL/200 μL) were added, and the sample was incubated for 1 h at 50°C. After cooling to room temperature, 0.5 mL of methanolic NaOH (1 M) was added, and the sample was evaporated under a stream of air at 50°C. The white crystals were reconstituted in 5 mL of water and extracted with TBME as described. The dried residues derived from the fraction of unconjugated steroids were derivatized employing 100 μL of MSTFA/NH_4_I/ethanethiol 1000/2/3 (v/w/v). After 20 min at 60°C, the samples were transferred into autosampler vials and forwarded to IRMS determinations.

The formerly glucuronidated and sulphated steroids were transferred to autosampler vials using 200 μL of MeOH, dried, and reconstituted in 100 μL of ACN/H_2_O (50/50, v/v) and forwarded to high performance liquid chromatography (HPLC)–based cleanup with fraction collection as described later on.

### 2.6 Sample Preparation Protocol for Metabolite Confirmation

All samples collected after the administration of nondeuterated MENT were processed similarly to routine sample preparation protocols [12, 13]. In contrast to the established method, 5 mL of urine was prepared. Based on the results obtained from the excretion study conducted with deuterated MENT, the sole focus was eventually on steroids excreted as glucuronic acid conjugates. These Phases I and II metabolites additionally represent the preferred target analytes for routine doping control analysis on exogenous steroids in most of the cases. All measurements for metabolite confirmation were conducted using HRMS. In order to determine potential detection windows for the novel metabolites, all samples were reprepared in‐line with the routine procedure employing 2 mL of urine and analyzed employing low resolution tandem mass spectrometry.

### 2.7 High Performance Liquid Chromatography–Based Cleanup

Due to the complex urinary biological matrix, additional cleanup of postadministration samples employing HPLC has been proven to be beneficial for metabolite identification [7, 8, 9, 10]. The method developed for MENT was closely related to the one employed during the metabolic studies on methylstenbolone [9]. All samples derived from the deuterated MENT administration trial were purified on an Agilent 1100 HPLC system (Waldbronn, Germany) equipped with a XBridge Shield RP18 5 mm (4.6 × 250 mm) column, protected by a XBridge Shield RP18 5 mm (4.6 × 20 mm) guard column from Waters (Eschborn, Germany). The gradient started with 20% ACN and 80% H_2_O, then to 100% ACN in 25 min, and hold for 8 min before re‐equilibration for 5 min at 20% ACN. The column flow was at 1 mL/min, and the injection volume was 100 μL. Fractions were collected using a FOXY R1 automatic fraction collector (Axel Semrau, Sprockhövel, Germany). The collection windows were adopted based on the known retention times for both endogenous steroids and the known metabolites of MENT in order to separate these from the rest of potential metabolites, as it was assumed that the already known metabolites will represent the most abundant ones after administration. Seven fractions were collected from 3.00 to 6.00 (I), from 6.01 to 11.00 (II), from 11.01 to 16.00 (III), from 16.01 to 18.00 (IV), from 18.01 to 23.00 (V), from 24.01 to 28.00 (VI), and from 28.01 to 33.00 min (VII). Fraction IV contained both M1 and M2 together with androsterone and etiocholanolone; unchanged MENT was found in fraction III.

All collected fractions were evaporated to dryness, derivatized as described for the fraction of unconjugated steroids (*vide supra*), and forwarded to hydrogen IRMS.

### 2.8 Gas Chromatography/Thermal Conversion/Isotope Ratio Mass Spectrometry

All measurements to determine the hydrogen isotope ratio (HIR) of potential urinary metabolites were conducted on a Delta V Plus IRMS (Thermo Fisher Scientific, Bremen, Germany) coupled to a Trace GC 1310 equipped with a TriPlusRSH Autosampler via the GC IsoLink CNH and the ConFlow IV (all Thermo). The thermal conversion ceramic tubing (320‐mm length, 0.5‐mm inner diameter, and 1.5‐mm outer diameter from IVA Analysentechnik, Meerbusch, Germany) was operated at 1450°C for pyrolysis. The IRMS was hyphenated to an ISQ mass spectrometer (Thermo) operated in positive electron ionization mode recording total ion chromatograms from *m/z* 50 to 800.

The GC column was selected to mimic the chromatographic conditions employed in routine, and therefore, a HP‐Ultra 1 column (length 17 m, i.d. 0.2 mm, film thickness 0.11 μm) from Agilent (Waldbronn, Germany) was installed. The temperature program was also based on the routine conditions and started at 180°C held for 2 min, then with 3°C/min to 250°C, and then with 40°C/min to the final temperature of 320°C, which was held for 3 min resulting in a total run time of 30 min. Injections were done in split mode with a split ratio of 5 at 280°C using the programmed flow option starting with 3 mL/min of helium (purity 5.0), held for 1.5 min, and then changed with a speed of 10–2.5 mL/min for the analytical run. The injection volume was 4 μL. Data were acquired using either Isodat 3.0 or Xcalibur 2.2 (both Thermo).

### 2.9 High‐Accuracy/High‐Resolution Mass Spectrometry

All high‐resolution measurements were carried out employing a Q Exactive GC Orbitrap GC‐MS/MS system (Thermo). The Trace 1310 GC was equipped with an Agilent HP‐Ultra 1 analytical column with similar dimensions as described, and the temperature program started at 180°C, increased at 3°C/min to 240°C and then with 40°C/min to 320°C, and held for 2 min resulting in a total run time of 24 min. Injections of 1 μL were performed in split mode at 300°C employing a split ratio of 1/5 and a purge flow of 5 mL/min using helium (purity grade 4.6). Constant pressure was set to 14.9 psi, and transfer line temperatures were at 300°C and 280°C for the first and second transfer line, respectively. Data were acquired either in full MS mode covering a scan range of *m/z* 100–700 or by parallel reaction monitoring (PRM) using an isolation window of *m/z* 1.3. Resolution was set to 60,000 (FWHM), and data were collected and evaluated using Xcalibur (Version 4.0, Thermo). Daily mass calibration of the instrument yielded mass accuracy in the range ± 2 ppm.

### 2.10 Multiple Reaction Monitoring Mass Spectrometry

The MRM measurements were conducted in parallel to the Cologne doping control laboratory routine setup employing a Thermo TSQ 8000 Evo mass spectrometer coupled to a Trace 1310 GC (Thermo). The analytical column was again an Agilent HP‐Ultra 1 using helium (purity grade 4.6) as carrier gas at a constant pressure of 15.1 psi. The oven temperature program started at 183°C and increase at 3°C/min to 232°C and then at 40°C/min to 310°C (2 min hold time). The injection volume was 1.5 μL, and a split ratio of 1/10 was applied. Injection temperature was at 300°C. Specific ion transitions were chosen for each analyte, and collision energies were optimized for each transition. All data were acquired and evaluated using Xcalibur (Version 4.0, Thermo).

### 2.11 Low‐Resolution Gas Chromatography/Mass Spectrometry

Deuteration of the acetylated MENT was monitored using an Agilent 7890 GC (Waldbronn, Germany) equipped with an Agilent J&W Scientific DB‐17MS column (length 30 m, i.d. 0.25 mm, film thickness 0.25 μm) and coupled to an Agilent 7000 triple quadrupole mass spectrometer. The initial oven temperature was 100°C, held for 1 min and increased at 40°C/min to 220°C, then at 10°C/min to 320°C, and finally held for 3 min. The inlet was at 280°C, and samples were injected in pulse splitless mode with a volume of 4 μL. Carrier gas was helium (purity 4.6) at a constant flow of 1.4 mL/min. Data were acquired in full scan mode ranging from *m/z* 80 to 500 and evaluated using Agilent Mass Hunter Qualitative Analysis (B.06.00).

## 3 Results and Discussion

Aim of this research project was to identify urinary metabolites of MENT, confirm these, and test for their suitability to be implemented into routine doping controls. Results will therefore be presented in this logical order followed by preliminary structural elucidations of the two most promising candidates for doping controls.

### 3.1 Urinary Metabolites of Deuterated MENT

**FIGURE 4:**
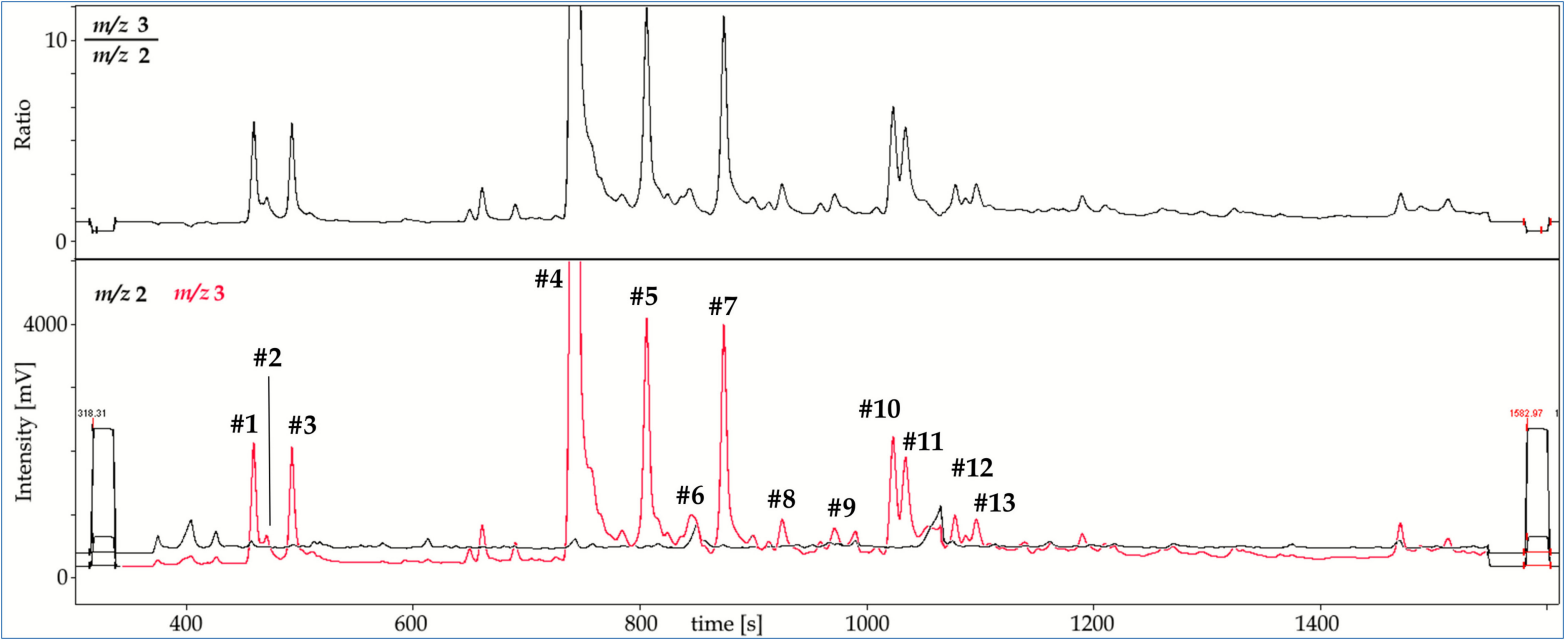
IRMS chromatogram obtained on the fraction of unconjugated steroids 12 h after the administration of 30 mg of sixfold deuterated MENT‐Ac. Each number represents a deuterated compound.

Already 1 h after oral application of MENT‐Ac, the first deuterated metabolites were visible in the unconjugated fraction of steroids in urine. Overall, 13 different metabolites (listed in Table S1) of MENT were excreted unconjugated, mainly during the first 36 h after application. An example of how HIR can aid in metabolite detection is shown in Figure 4. Each peak in the chromatogram belongs to a deuterated metabolite of MENT. Regarding the early eluting compounds around 500 s, these may represent degradation products of thermally labile metabolites considering the fact that these compounds only retained one oxygen atom. In general, MENT underwent the expected metabolic transformations including mainly hydroxylation and reductions at different sites of the steroidal backbone. Metabolite 8 exhibiting a molecular ion at *m/z* 494.3182 translating into an elemental composition of C_27_H_42_D_4_O_4_Si_2_ was suggested to be a direct metabolite of the administered MENT‐Ac. After the oral administration of an esterified steroid, it is expected that the steroid is saponified before entering the circulation and undergoing all Phases I and II metabolic conversions. A small amount of MENT‐Ac appears to be resorbed, reduced, hydroxylated, and excreted into urine unconjugated during the first day after administration, representing a metabolite of the first path effect.

Approximately half of the metabolites excreted unconjugated were additionally found in the fraction of glucuroconjugated steroids. In total, more than 40 metabolites of the deuterated MENT‐Ac were detectable in this fraction (Table S1). Metabolites excreted sulfoconjugated were scarce for MENT and did not show any prolonged detectability. Based on this finding and considering that in routine doping controls mainly unconjugated and glucuronidated steroid metabolites are considered, only these two fractions were further investigated here.

### 3.2 Confirmed Metabolites Employing Nondeuterated MENT

Deuterated metabolites of a steroidal compound are not only straightforwardly detectable by IRMS; their presence is also relatively easy confirmed using HRMS due to the unique mass of the deuterium atom [7, 8, 9, 10]. The biological background or, regarding mass spectrometry, the analytical noise for these compounds is therefore often neglectable. This may result in a very sensitive detection of these metabolites, even if they are only present in urine in the pg/mL range and may result in very long detection times, presumably much longer than their nondeuterated counterparts [9, 14]. Additionally, steroid metabolism always shows interindividual variations, and a metabolite easily detectable in one individual may be hampered by endogenous coelutions in another individual or may even not be present. Therefore, three additional volunteers administered nondeuterated MENT‐Ac in order to confirm the metabolites identified using deuterated MENT‐Ac.

**FIGURE 5:**
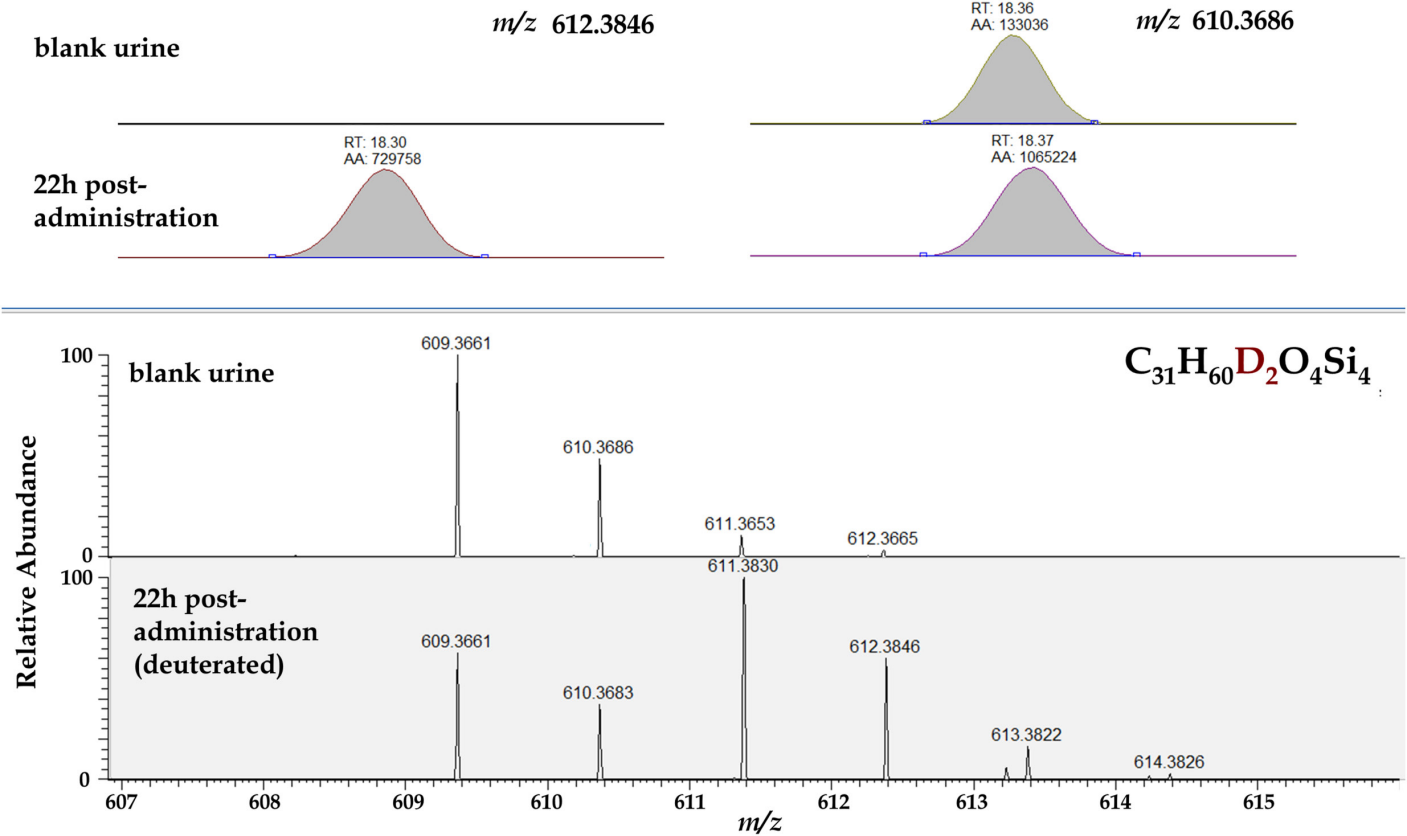
Upper part: HRMS extracted ion chromatograms (± 20 ppm) of a novel metabolite of MENT, left side deuterated, and right side nondeuterated. Lower part: Corresponding HRMS mass spectra. Further information in the text.

From the *n* = 50 metabolites attributable to deuterated MENT‐Ac, *n* = 28 were present in all four volunteers at significant amounts, that is, with urinary concentrations high enough to be potentially implemented into routine doping control analytical procedures. After a first careful inspection of extracted ion chromatograms, molecular masses, and related fragmentation pathways, half of the found metabolites were excluded from further investigations due to significant coelutions as already described for M1 and M2 (Figure 1). Exemplarily for the newly detected metabolites, this phenomenon is also shown in Figure 5. The extracted ion chromatogram for the deuterated compound at *m/z* 612 does not exhibit interfering signals in the blank urine sample, and this (deuterated) metabolite was detectable for up to 143 h after administration. Considering the nondeuterated administration trials, in none of the volunteers, the preadministration urine sample was blank, showing at least one endogenous substance coeluting with an interfering mass (considering the nondeuterated metabolite), retention time, and fragmentation pattern typical for trimethylsilylated (TMS) compounds. As shown in Figure 5, the ^13^C isotopologue of *m/z* 609 present at *m/z* 610.3686 shows exactly the same *m*/*z* as the nondeuterated metabolite. An additional interfering compound was also detected after the administration of the deuterated MENT present at *m*/*z* 611.3830 (Figure 5, lower part). This is not the mono‐deuterated metabolite, which would show an ion at *m/z* 611.3782. These coelutions were a common problem identified for human urinary metabolites of MENT resulting in only *n* = 14 metabolites further investigated here and listed in Table 1.

**TABLE 1:** All metabolites of MENT further investigated regarding their potential to aid sport drug testing.

Metabolite	Retention time [min]	Molecular ion (*m/z*)	Elemental composition	Detection time in hours (V_1, V_2, V_3)
M2	10.30	432.2878	C_25_H_44_O_2_Si_2_	NA
M1	11.22	434.3029	C_25_H_46_O_2_Si_2_	NA
M522	12.42	522.3385	C_28_H_54_O_3_Si_3_	44, 36, 43
M518_2	12.64	518.3068	C_28_H_50_O_3_Si_4_	49, 48, 49
MENT	12.73	432.2880	C_25_H_44_O_2_Si_2_	23, 33, 43
M518_1	13.20	518.3068	C_28_H_50_O_3_Si_4_	36, 68, 56
M6	13.96	516.2914	C_28_H_48_O_3_Si_4_	30, 44, 28
M518_3	14.49	518.3068	C_28_H_50_O_3_Si_4_	45, 60, 56
M3	14.71	608.3559	C_31_H_60_O_4_Si_4_	45, 60, 68
M4	15.84	522.3383	C_28_H_54_O_3_Si_3_	28, 30, 28
M5	15.95	610.3720	C_31_H_62_O_4_Si_4_	68, 92, 68
M8	17.12	604.3252	C_31_H_56_O_4_Si_4_	24, 44, 30
M10	17.61	578.3103	C_29_H_54_O_4_Si_4_	92, 82, 68
M11	17.70	606.3432	C_31_H_58_O_4_Si_4_	28, 33, 28

### 3.3 Selectivity and Detection Windows of All Investigated Metabolites

**FIGURE 6:**
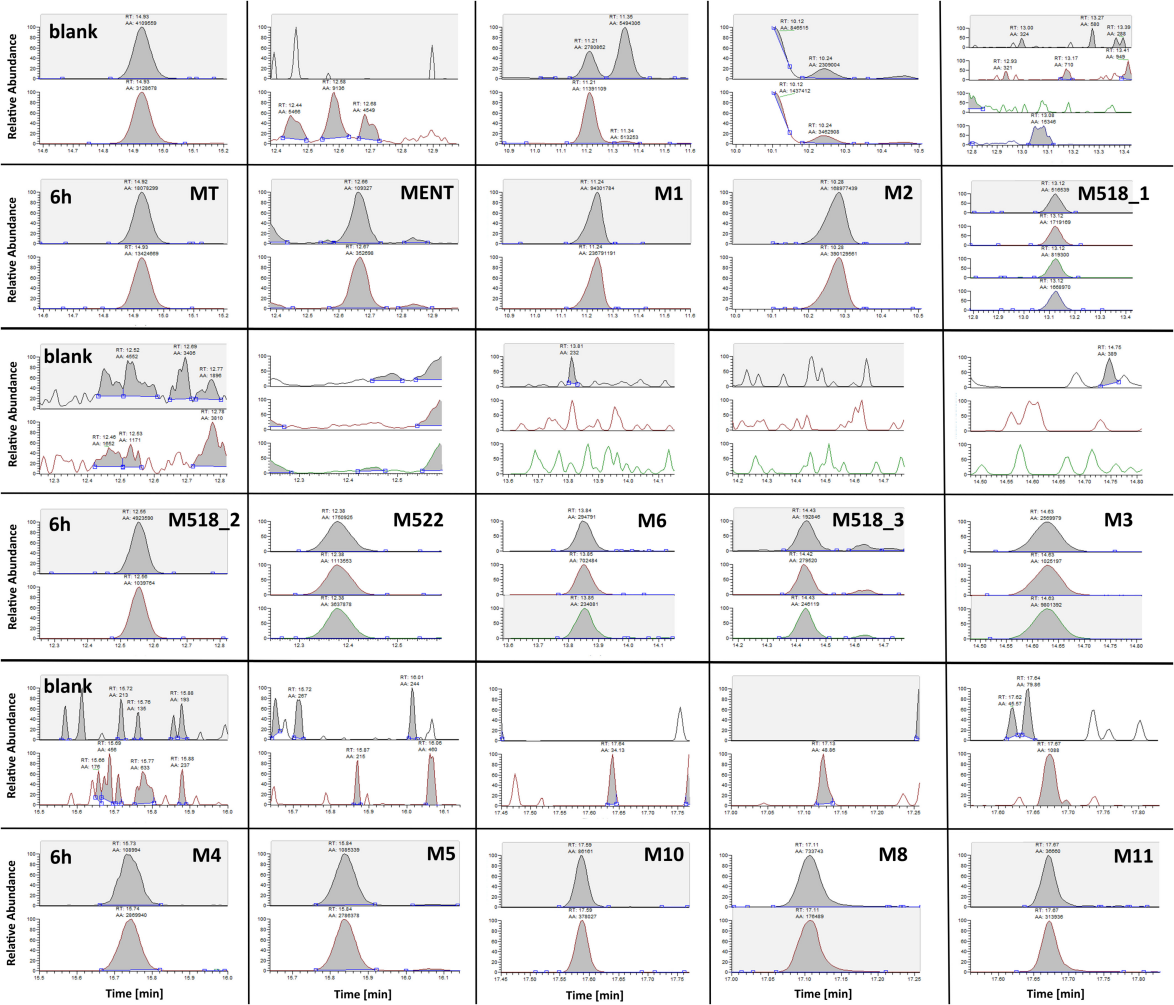
MRM‐chromatograms obtained for the 14 metabolites of MENT and methyltestosterone (MT) as internal standard. The top row in each case shows the preadministration sample (blank) and the bottom row the sample collected 6‐h postadministration. The respective MRM transitions are listed in Table 2.

As the aim of this research project was not only to detect novel metabolites of MENT but to identify those beneficial for sports drug testing, each metabolite was investigated regarding its potential to be implemented into routine doping controls. A general overview over the obtained chromatographic conditions and the employed MRM transitions is given in Figure 6 and Table 2.

**TABLE 2:** MRM‐transitions and collision energies for all investigated MENT‐metabolites; the dwell time was uniformly set to 30 ms.

Time (min)	Scan name	Precursor ion (*m/z*)	Product ion (*m/z*)	Collision energy (eV)
10.1	M1	432.3	417.3	8
	M1	417.3	327.2	8
	M2	419.3	329.2	8
	M2	329.2	239.2	8
12.0	MENT	417.3	133.1	16
	MENT	417.3	223.2	9
	M518_2	518.3	218.2	18
	M518_2	518.3	269.2	13
	M518_2/M518_1	518.3	323.2	13
	M518_2/M518_1	518.3	413.3	8
	M522	507.3	327.2	8
	M522	522.3	195.1	25
	M522	522.3	327.2	10
13.6	MeT	446.3	301.3	11
	MeT	301.3	169.1	7
	M6	516.3	243.2	25
	M6	411.3	243.2	10
	M6	516.3	321.3	25
	M518_3	413.3	398.3	13
	M518_3	503.3	219.2	18
	M518_3	503.3	398.3	18
	M3	593.3	413.3	8
	M3	608.3	503.3	20
	M3	608.3	593.3	8
15.2	M4	417.3	241.2	23
	M4	507.3	255.2	13
	M5	595.3	415.3	8
	M5	610.3	595.3	8
16.5	M10	578.3	327.3	13
	M10	578.3	517.3	8
	M8	589.3	243.2	18
	M8	604.3	243.2	25
	M11	591.3	333.3	8
	M11	606.3	591.3	8

#### 3.3.1 MENT

Depending on the chosen ion transition, it is possible to use MENT (excreted as glucuroconjugate) as a target analyte to detect the misuse of MENT and MENT‐Ac. The main drawback of MENT is its relatively short detection window of only 23–43 h after administration as shown in Table 1 for all three volunteers.

#### 3.3.2 M1 and M2

**FIGURE 7:**
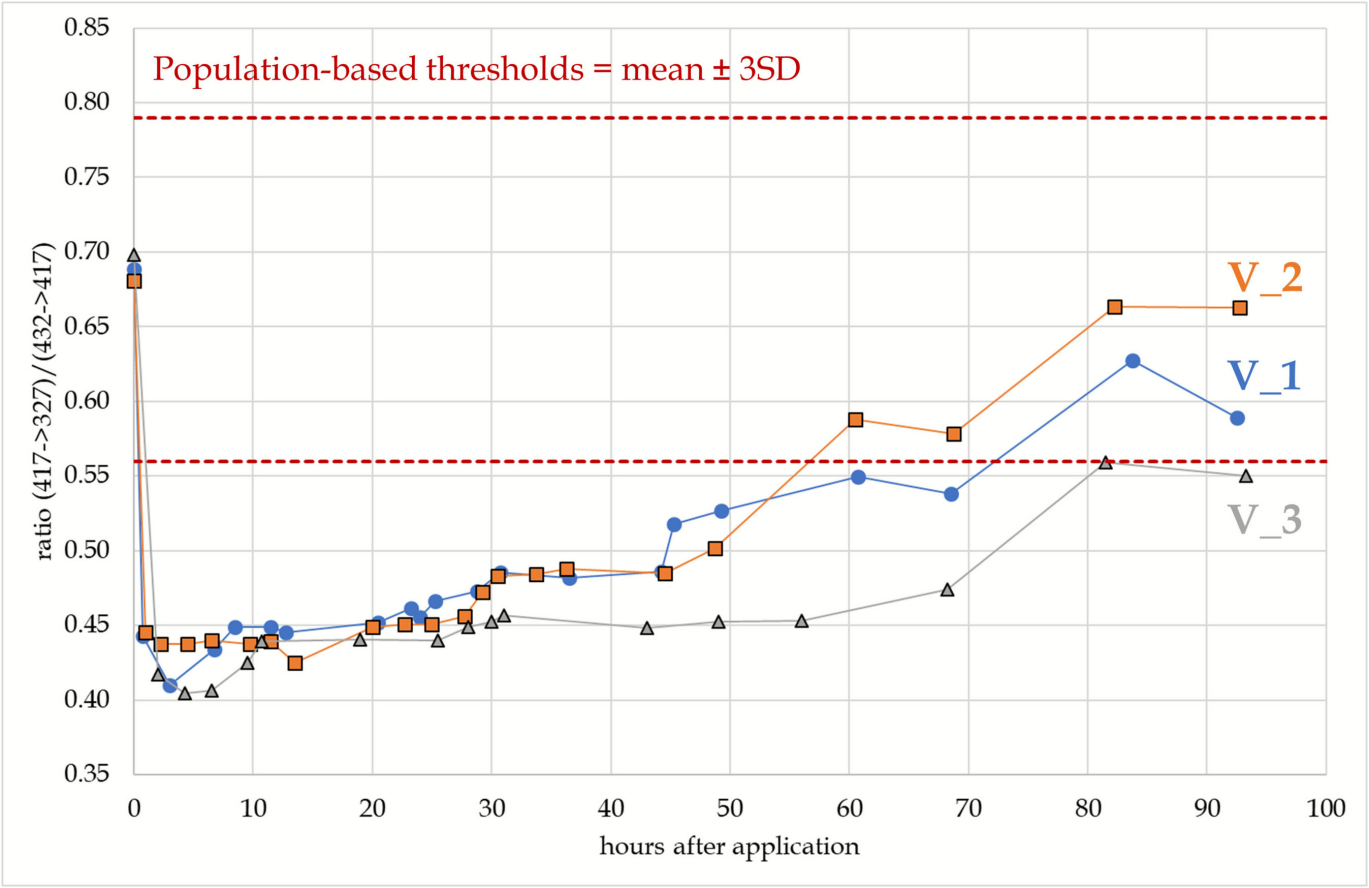
Shift observed for M2 in the ion transition ratio build by dividing the arear under the peak of *m/z* 417–327 by *m/z* 432–417 after the administration of 30 mg of MENT‐Ac. Further information in the text.

Not only in the blank urine samples collected in the four volunteers participating in the administration trials but also in all of the 200 investigated athlete routine samples, interfering compounds appearing at the retention times of M1 and M2 were visible under the chromatographic conditions (Figures 1 and 6). Considering the presence of the same diagnostic ions demonstrates that the endogenous coelutions seems to be structurally related to M1 and M2, but neither of these fulfilled the positivity criteria for MENT metabolites when applying the identification criteria as per TD2023IDCR issued by WADA [15]. Discrete precursor/product ion pairs and/or their relative abundances differentiating between the endogenous coelution and M1 and M2 of MENT were sought, and although for M1 preadministration and postadministration samples did not show a significant difference, M2 could be identifiable as demonstrated exemplarily in Figure 7. Here, the ion transition ratio of the areas under the peak for the transitions *m/z* 417–327 and *m/z* 432–417 is plotted over time in the samples obtained in the nondeuterated excretion studies. A pronounced shift in the found ion transition ratio is visible, starting from 0.69 preadministration to values between 0.40 and 0.45 postadministration. In order to elucidate if this shift was robust, the same ion transition ratio was evaluated for the endogenous coelution in the investigated athlete population encompassing *n* = 200 individuals. A preliminary population‐based threshold for the endogenous coelution was calculated by adding the threefold standard deviation to the mean value in line with the recommendations of the International Federation of Clinical Chemistry for Gaussian distributed data [16]. As shown in Figure 7, the ratio was found outside the population‐defined range for approximately 50 h postadministration enabling, in principle, the detection of MENT misuse. Under routine doping control conditions, application of this ion transition ratio might be suboptimal as the defined ratio depends on the individual mass spectrometer and may even be affected by intensity shifts over time on the same instrument. Therefore, other novel metabolites may be more promising here.

#### 3.3.3 M518_1, M518_2, M518_3, M_522, M4, and M6

Hydroxylation is very common during the Phase I metabolism of steroids in humans, and this obviously holds true for MENT, too. Several metabolically mono‐hydroxylated compounds were identified in postadministration urine samples, resulting in three oxygen atoms per metabolite. All of these metabolites have not been reported before. Identifying the site of hydroxylation is difficult when solely chromatographic and mass spectrometric data exist. The majority of fragment ions were related to the common fragmentation scheme for trimethylsilylated steroids, that is, comprising mainly losses of 15 or 90 u. The only information that might be helpful here was the number of remaining deuterium atoms in each metabolite. For example, M518_1 comprised four deuterium atoms, whereas in M518_2 and M518_3, only three deuterium atoms were found. This suggests for the latter ones a more likely location of the hydroxylation at the A‐ or B‐ring, whereas for M518_1, the D‐ring was the potential site for Phase I metabolism. Depending on the individual metabolism, all of these novel metabolites were detectable for 2–3 days postadministration (Table 1).

#### 3.3.4 M3, M8, and M11

The structure of these bis‐hydroxylated novel metabolites was also not straightforwardly deducible from the obtained mass spectrometric data. As expected for polar metabolites, they were mainly produced and excreted in the early post administration phase and therefore detectable only for 1 or 2 days (Table 1). Only M3 was excreted for up to 3 days postadministration and may by structurally comparable to M5, identified as one of the most promising candidates for sports drug testing together with M10.

#### 3.3.5 M5 and M10

**FIGURE 8:**
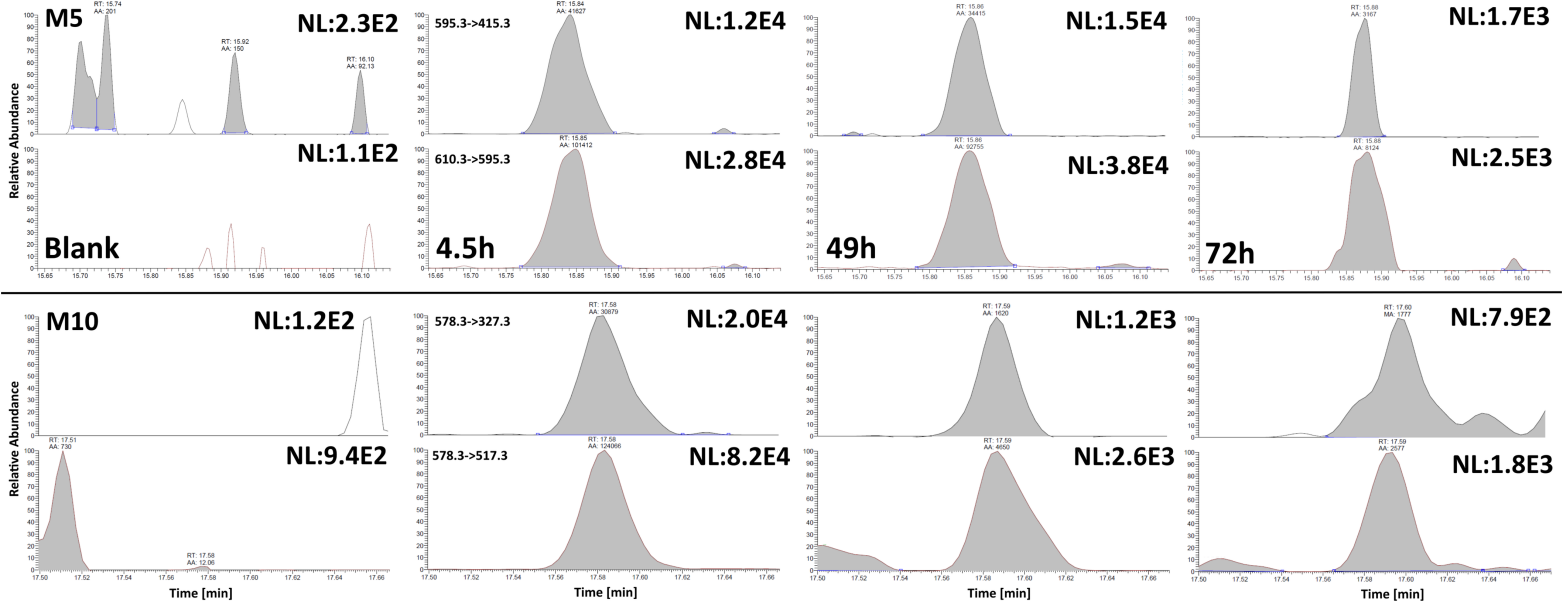
MRM chromatograms obtained for M5 (upper part) and M10 (lower part) at different time point preadministration and postadministration. The normalization value (NL) is also given to allow for intensity estimation.

Both metabolites showed comparable and promising results as potential markers for MENT administrations under routine conditions and were detectable for 3–4 days despite also representing the class of bis‐hydroxylated compounds (Table 1). Chromatograms obtained preadministration and postadministration are depicted in Figure 8 to demonstrate the suitability of both metabolites. In none of the *n* = 200 athlete routine samples, a significant coelution was detectable for M5 and M10.

### 3.4 Preliminary Structural Elucidation of M5 and M10

Considering the accurate masses obtained for both metabolites, hydroxylation was again the predominant Phase I metabolic reaction to increase their polarity. As both metabolites were found to be substantially different in their mass spectrometric behavior, a dedicated discussion for each substance appeared warranted. Of note, for both metabolites, the shown structures can only be considered as tentative. Further investigations into the structure of both metabolites may be warranted in the future if these demonstrate to be useful markers in routine doping controls.

#### 3.4.1 Metabolite M5

**FIGURE 9:**
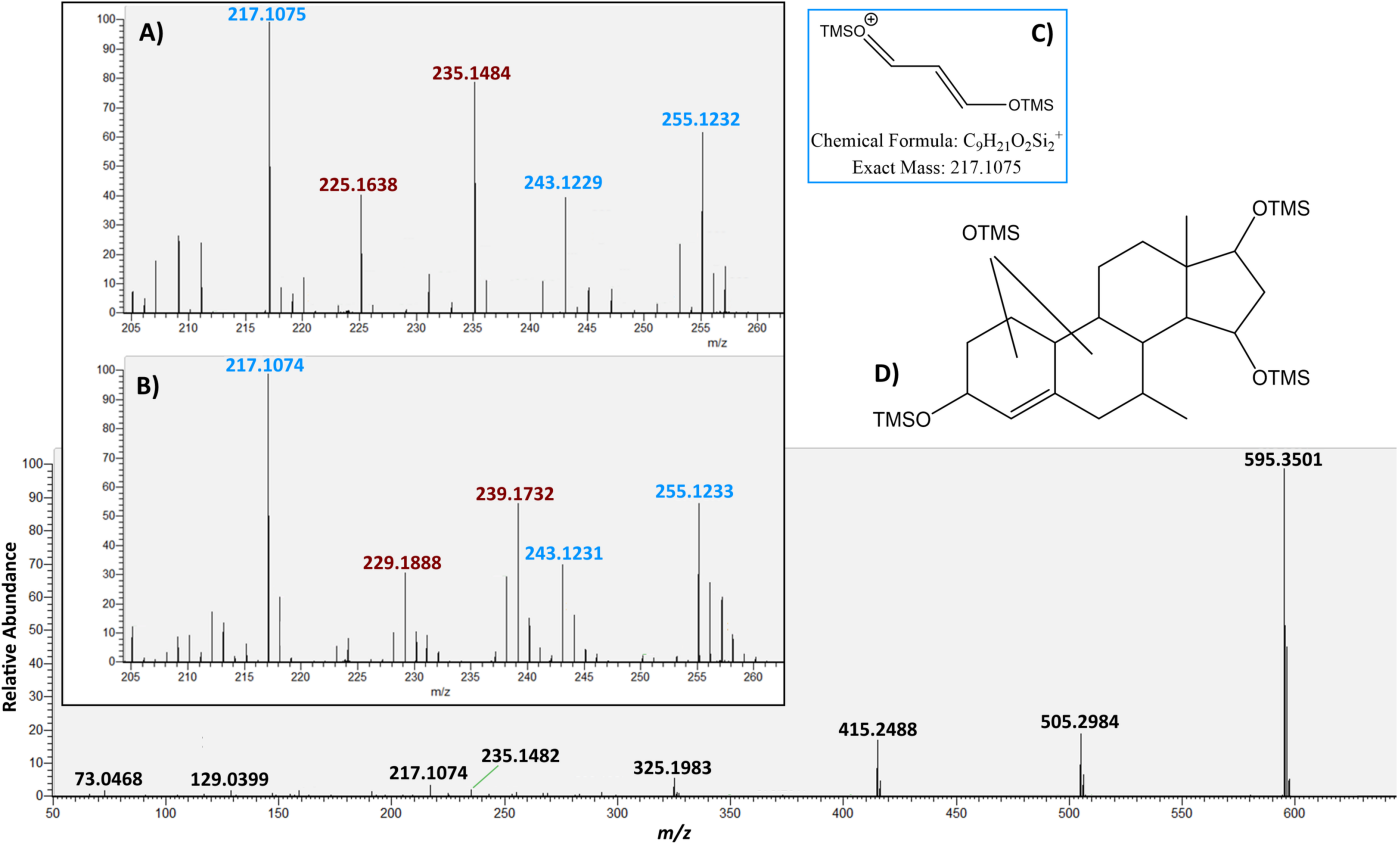
MS/MS spectrum of the molecular ion of M5 at *m/z* 610.3720 (lower part) and a magnification of the part between *m/z* 205 and 260 for both the native (A) and the fivefold deuterated (B) metabolite. The proposed structure of the relevant fragment at *m/z* 217.1075 (C) and the entire metabolite (D) are shown. Further information in the text.

M5 was found to be fivefold deuterated with accurate molecular masses of *m/z* 610.3720 for the nondeuterated and *m/z* 615.4031 for the deuterated tetrakis‐TMS derivatives. The high‐resolution mass spectrum of the native metabolite is shown in Figure 9 (lower part) and mainly comprises the common fragments described for trimethylsilylated steroids with neutral losses of 15 and 90 u. No structural details can be deduced by the interpretation of these fragments. However, a comparison of the fragments of the deuterated and the native metabolite in the range between *m/z* 200 and *m/z* 260 allows for some interpretations (Figure 9A,B). The D‐ring fragments (in blue) do not show any deuterium‐based mass shift, whereas fragments assigned to the A‐ and B‐ring (in red) are affected by deuterium labeling. The determined accurate masses and accordingly deduced elemental compositions of the D‐ring fragments at *m/z* 255 (C_12_H_23_O_2_Si_2_^+^), *m/z* 243 (C_11_H_23_O_2_Si_2_^+^), and *m/z* 217 (C_9_H_21_O_2_Si_2_^+^) support the presence of 2 oxygen atoms in the D‐ring, with the fragment at *m/z* 217 shown in Figure 9C being reportedly characteristic for C17 and C15 bis‐hydroxylated steroids [17, 18]. Regarding the location of the additional oxygen atom, no further information can be derived from the mass spectrometric experiments; however, the most probable site for hydroxylation is in the A or B ring resulting in the preliminarily structure for M5 supposed in Figure 9D.

#### 3.4.2 Metabolite M10

**FIGURE 10:**
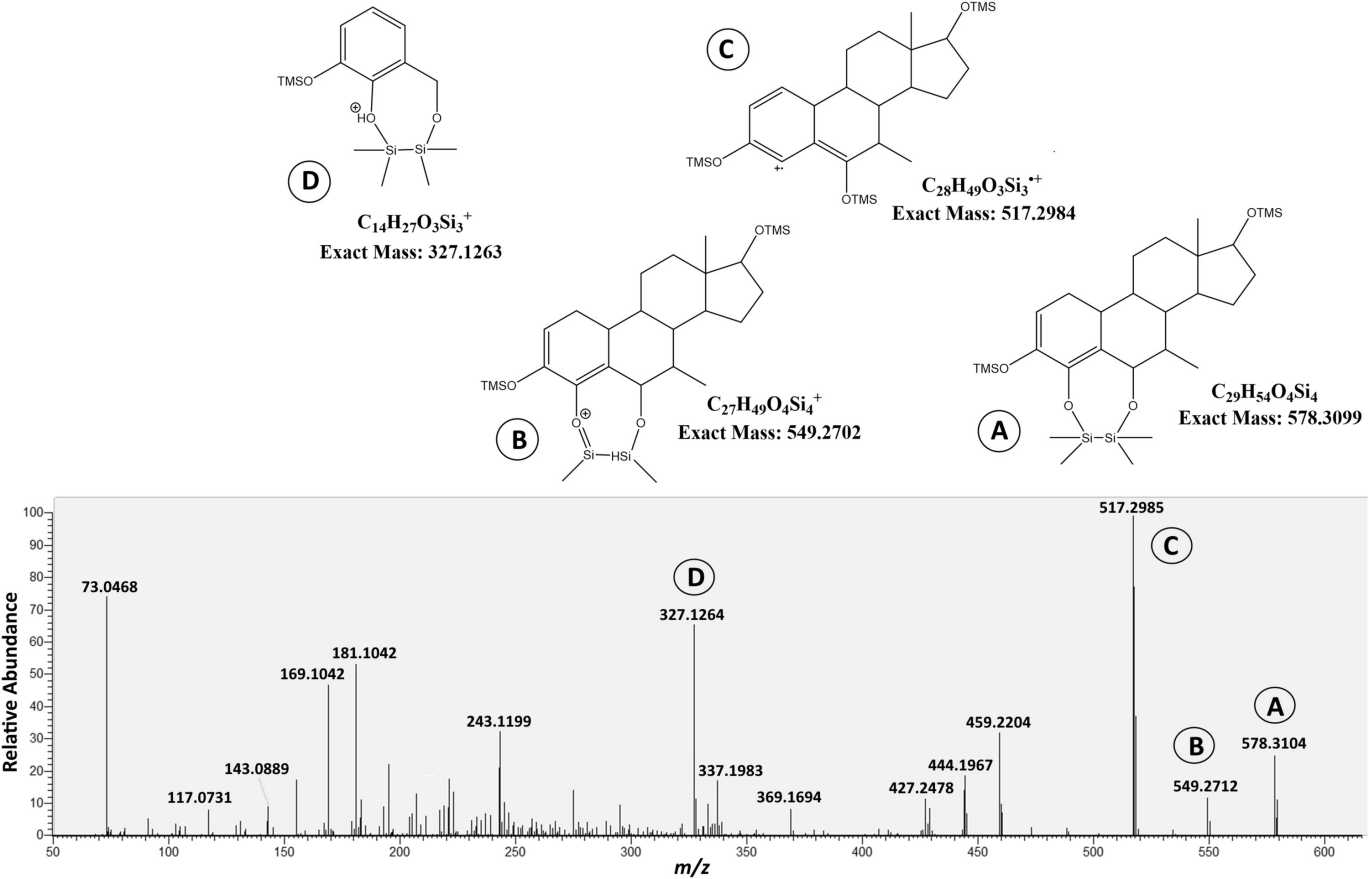
MS/MS spectrum of the pseudo‐molecular ion of M10 at *m/z* 578.3104 (lower part) together with hypothetically derived structures of relevant fragments.

In contrast to the very common fragmentation pattern found for M5, M10 showed an unexpected mass spectrometric behavior for a steroidal compound as shown in Figure 10. The expected molecular mass of *m/z* 608.3563 representing an elemental composition of C_31_H_60_O_4_Si_4_ in parallel to the other bis‐hydroxylated metabolites was not detectable. The pseudo‐molecular ion was found at *m/z* 578.3105 (C_29_H_54_O_4_Si_4_) displaying a nominal loss of −30 u (C_2_H_6_) as shown in Figure 10A. Although losses of 15 u (CH_3_), 28 u (C_2_H_4_), and even 29 u (C_2_H_5_) have been described for trimethylsilylated steroids, information on this loss of 30 u seems to be scarce [17, 18, 19]. It has been found for androst‐4‐en‐19‐ol‐3,17‐dione (19‐TMS), which cannot be related to the 19‐nor‐steroid MENT and for estra‐1,3,5(10)‐triene‐3,4‐diol‐17‐one‐4‐methyl ether (3‐TMS) [19]. The latter was taken as basis for further literature review, revealing that not only vicinal bis‐hydroxylated steroids containing a methyl ether but also bis‐hydroxylated‐bis‐TMS compounds may show the loss of 30 u [18, 20]. The position of the additional hydroxy‐functional groups in M10 could not be derived from the mass spectrometric data; besides, they should be placed in the A and B rings considering that the metabolite only contains three deuterium atoms (Figure 10B–D). The structure shown in Figure 10A represents only one potential hypothesis to demonstrate the idea explaining this unique fragmentation for trimethylsilylated steroids. The shown fragments (Figure 10B–D) were then derived from the pseudo‐molecular ions in agreement with the accurate masses found for both the deuterated and the nondeuterated metabolite. If the initial loss of 2 methyl groups represents an artifact of the derivatization or may be due to thermal instability during the chromatographic separation or occurs during electron ionization could not be clarified.

## 4 Conclusions

The human metabolism on MENT was reinvestigated to identify urinary metabolites better suitable for doping controls compared to the already described ones. This was accomplished by the administration of deuterated MENT‐Ac to one volunteer enabling the detection of 50 different metabolites comprising the deuterium label. In a second step, the found metabolites were verified to be present and detectable interindividually by additional administration trials with nondeuterated MENT‐Ac encompassing three male volunteers. From the *n* = 28 metabolites detectable in all volunteers, *n* = 14 was further investigated regarding their potential as routine doping control markers for MENT misuse. From the already described metabolites of MENT, only the parent compound seems to be a suitable marker under routine conditions. The 11 new metabolites can all be used in the context of sports drug testing with M5 and M10 offering the longest detection time after a single oral administration of MENT‐Ac. The structural elucidation of these metabolites (and all other novel metabolites) was found to be highly complicated, as the majority of fragmentation pathways was driven by the common TMS cleavages and, in general, no information about specific fragments of 7‐methylated steroid is at hand. Additionally, the synthesis of reference material to substantiate the hypothetically derived structures will be complicated as 7‐methylated steroid starting material is scarce. A reasonable and practical alternative will be the implementation of the novel metabolites in routine doping control methods and the usage of excretion study samples as quality controls for confirmation purposes.

## Funding

This study was supported by the World Anti‐Doping Agency (23A07MT), the Manfred‐Donike Institute for Doping Analysis, and the Bundesministerium des Innern, für Bau und Heimat.

## Conflicts of Interest

The authors declare no conflicts of interest.

## Supporting information


**Table S1:** All potential metabolites of deuterated MENT‐Ac found after a single oral administration of 30 mg. The given fraction represents unconjugated steroids (F), glucuronidated steroid (G) and sulphated metabolites (S), the roman numbers stand for the HPLC‐fraction each metabolite was found in.

## Data Availability

The data that support the findings of this study are available from the corresponding author upon reasonable request.

## References

[1] S. C. Lyster and G. W. Duncan , “Anabolic, Androgenic and Myotropic Activities of Derivatives of 7α‐Methyl‐19‐Nortestosterone,” Acta Endocrinologica 43 (1963): 399–411, 10.1530/acta.0.0430399. 13931986

[2] A. Segaloff , J. B. Weeth , M. Cuningham , and K. K. Meyer , “Hormonal Therapy in Cancer of the Breast XXIII. Effect of 7α‐Methyl‐19‐Nortestosterone Acetate and Testosterone Propionate on Clinical Course and Hormonal Excretion,” Cancer 17 (1964): 1248–1253, 10.1002/1097-0142(196410)17:10<1248::AID-CNCR2820171005>3.0.CO;2-A. 14236756

[3] J. Suvisaari , K. Sundaram , G. Noe , et al., “Pharmacokinetics and Pharmacodynamics of 7a‐Methyl‐19‐Nortestosterone After Intramuscular Administration in Healthy Men,” Human Reproduction 12 (1997): 967–973, 10.1093/humrep/12.5.967. 9194649

[4] E. Nieschlag , N. Kumar , and R. Sitruk‐Ware , “7α‐Methyl‐19‐Nortestosterone (MENTR): The Population Council's Contribution to Research on Male Contraception and Treatment of Hypogonadism,” Contraception 87 (2013): 288–295, 10.1016/j.contraception.2012.08.036. 23063338

[5] A. K. Agarwal and C. Monder , “In Vitro Metabolism of 7a‐Methyl‐19‐Nortestosterone by Rat Liver, Prostate, and Epididymis,” Endocrinology 123 (1988): 2187–2193, 10.1210/endo-123-5-2187. 2971524

[6] R. Kazlauskas , “Miscellaneous Projects for 2003,” in Recent Advances in Doping Analysis (12), eds. W. Schänzer , H. Geyer , A. Gotzmann , and U. Mareck (Sport und Buch Strauß, 2004), 261–268.

[7] M. Thevis , T. Piper , S. Horning , D. Juchelka , and W. Schänzer , “Hydrogen Isotope Ratio Mass Spectrometry and High‐Resolution/High‐Accuracy Mass Spectrometry in Metabolite Identification Studies: Detecting Target Compounds for Sports Drug Testing,” Rapid Communications in Mass Spectrometry 27 (2013): 1904–1912, 10.1002/rcm.6648. 23939956

[8] T. Piper , W. Schänzer , and M. Thevis , “Revisiting the Metabolism of 19‐Nortestosterone Using Isotope Ratio and High Resolution/High Accuracy Mass Spectrometry,” Journal of Steroid Biochemistry and Molecular Biology 162 (2016): 80–91, 10.1016/j.jsbmb.2015.12.013. 26699683

[9] T. Piper , G. Fusshöller , W. Schänzer , A. Lagojda , D. Kuehne , and M. Thevis , “Studies on the In Vivo Metabolism of Methylstenbolone and Detection of Novel Long Term Metabolites for Doping Control Analysis,” Drug Testing and Analysis 11 (2019): 1644–1655, 10.1002/dta.2736. 31733090

[10] T. Piper and M. Thevis , “Investigations Into the Human Metabolism of Ecdysterone,” Drug Testing and Analysis 15 (2023): 1503–1520, 10.1002/dta.3582. 37778393

[11] L. Dehennin , A. Reiffsteek , and R. Scholler , “Simple Methods for the Synthesis of Twenty Different, Highly Enriched Deuterium Labelled Steroids, Suitable as Internal Standards for Isotope Dilution Mass Spectrometry,” Biomedical Mass Spectrometry 7 (1980): 493–499, 10.1002/bms.1200071108.

[12] U. Mareck , H. Geyer , G. Opfermann , M. Thevis , and W. Schänzer , “Factors Influencing the Steroid Profile in Doping Control Analysis,” Journal of Mass Spectrometry 43 (2008): 877–891, 10.1002/jms.1457. 18570179

[13] M. Thevis , G. Fusshöller , and W. Schänzer , “Zeranol: Doping Offence or Mycotoxin? A Case Related Study,” Drug Testing and Analysis 3 (2011): 777–783, 10.1002/dta.352. 22095651

[14] T. Piper , G. Fusshöller , W. Schänzer , and M. Thevis , “Investigations on the In Vivo Metabolism of 5α‐Androst‐2‐En‐17‐One,” Rapid Communications in Mass Spectrometry 36, no. 17 (2022): e9343, 10.1002/rcm.9343. 35737649

[15] WADA Technical Document – TD2023IDCR , “Minimum Criteria for Chromatographic‐Mass Spectrometric Confirmation of the Identity of Analytes for Doping Control Purposes,” accessed July 11, 2025, https://www.wada‐ama.org/sites/default/files/2023‐02/td2023idcrv1.1_eng_final.pdf.

[16] H. E. Solberg , “Approved Recommendation (1987) on the Theory of Reference Values. Part 5. Statistical Treatment of Collected Reference Values. Determination of Reference Limits,” Clinica Chimica Acta 170 (1987): S13–S32, 10.1016/0009-8981(87)90151-3.

[17] G. von Unruh and G. Spiteller , “Schlüsselbruchstücke und Schlüsseldifferenzen als Kriterium bei der Datenerfassung von Massenspektren,” Tetrahedron 26 (1970): 3303–3311, 10.1016/S0040-4020(01)92885-6.

[18] D. J. Harvey and P. Vouros , “Mass Spectrometric Fragmentation of Trimethylsilyl and Related Alkylsilyl Derivatives,” Mass Spectrometry Reviews 39 (2020): 105–211, 10.1002/mas.21590. 31808199

[19] H. L. J. Makin , D. J. H. Trafford , and J. Nolan , Mass Spectra and GC Data of Steroids: Androgens and Estrogens (Wiley‐VCH, 1999).

[20] S. E. Hattox and R. C. Murphy , “Mass Spectrometry and Gas Chromatography of Trimethylsilyl Derivatives of Catecholamine Related Molecules,” Biological Mass Spectrometry 5 (1978): 338–345, 10.1002/bms.1200050505. 656558

