# Supplementary material for: Investigations Into the Human Metabolism of Trestolone (7α‐Methyl‐19‐Nortestosterone)
Source: Drug Test Anal. 2025 Dec 17;18(3):341–53. doi: 10.1002/dta.70018 (PMC12983052; doi:10.1002/dta.70018)
Supplement: Supplementary file 1 — Table S1: All potential metabolites of deuterated MENT‐Ac found after a single oral administration of 30 mg. The given fraction represents unconjugated steroids (F), glucuronidated steroid (G) and sulphated metabolites (S), the roman numbers stand for the HPLC‐fraction each metabolite was found in. [file DTA-18-341-s001.docx]

Investigations into the human metabolism of Trestolone (7α-methyl-19-nortestosterone)

Thomas Piper, Gregor Fusshöller, Mario Thevis

*Supporting Information*

Table S1: All potential metabolites of deuterated MENT-Ac found after a single oral administration of 30 mg. The given fraction represents unconjugated steroids (F), glucuronidated steroid (G) and sulphated metabolites (S), the roman numbers stand for the HPLC-fraction each metabolite was found in.

|  | **Retention time [min]** | **Molecular ion *[m/z]*** | **Elemental composition** | **Fraction** |
| --- | --- | --- | --- | --- |
| #1 | 5.50 | 346.2624 | C_22_ H_30_ D_4_ O Si | F, G_IV |
| #2 | 5.70 | 346.2624 | C_22_ H_30_ D_4_ O Si | F, G_IV |
| #3 | 6.00 | 346.2624 | C_22_ H_30_ D_4_ O Si | F, G_IV |
| #4 | 9.51 | 437.3188 | C_25_ H_39_ D_5_ O_2_ Si_2_ | F, G_III |
| #5 | 10.45 | 437.3188 | C_25_ H_39_ D_5_ O_2_ Si_2_ | F, G_III |
| #6 | 11.37 | 432.2805 | C_25_ H_36_ D_4_ O_2_ Si_2_ | F |
| #7 | 11.46 | 434.2970 | C_25_ H_38_ D_4_ O_2_ Si_2_ | F |
| #8 | 12.29 | 494.3182 | C_27_ H_42_ D_4_ O_4_ Si_2_ | F |
| #9 | 13.75 | 612.3835 | C_31_ H_60_ D_2_ O_4_ Si_4_ | F |
| #10 | 14.68 | 521.3255 | C_28_ H_47_ D_3_ O_3_ Si_3_ | F, G_III |
| #11 | 14.96 | 614.3991 | C_31_ H_60_ D_3_ O_4_ Si_4_ | F |
| #12 | 17.14 | 685.3955 | C_33_ H_65_ D_2_ O_5_ Si_5_ | F |
| #13 | 18.17 | 688.4183 | C_33_ H_66_ D_3_ O_5_ Si_5_ | F |
| #14 | 13.71 | 612.3836 | C_31_ H_60_ D_2_ O_4_ Si_4_ | G_I |
| #15 | 14.89 | 614.3993 | C_31_ H_60_ D_3_ O_4_ Si_4_ | G_I |
| #16 | 10.29 | 524.3475 | C_28_ H_48_ D_4_ O_3_ Si_3_ | G_II, S_II |
| #17 | 11.37 | 432.2812 | C_25_ H_36_ D_4_ O_2_ Si_2_ | G_II |
| #18 | 11.53 | 524.3475 | C_28_ H_48_ D_4_ O_3_ Si_3_ | G_II |
| #19 | 11.71 | 435.3032 | C_25_ H_37_ D_5_ O_2_ Si_2_ | G_II |
| #20 | 12.04 | 524.3476 | C_28_ H_48_ D_4_ O_3_ Si_3_ | G_II |
| #21 | 13.57 | 522.3315 | C_28_ H_46_ D_4_ O_3_ Si_3_ | G_II |
| #22 | 16.73 | 615.4051 | C_31_ H_61_ D_3_ O_4_ Si_4_ | G_II |
| #23 | 17.09 | 612.3833 | C_31_ H_60_ D_2_ O_4_ Si_4_ | G_II |
| #24 | 17.58 | 598.3656 | C_30_ H_52_ D_5_ O_4_ Si_4_ | G_II |
| #25 | 18.36 | 612.3846 | C_31_ H_60_ D_2_ O_4_ Si_4_ | G_II |
| #26 | 10.03 | 434.2966 | C_25_ H_38_ D_4_ O_2_ Si_2_ | G_III |
| #27 | 10.51 | 466.3227 | C_26_ H_42_ D_4_ O_3_ Si_2_ | G_III |
| #28 | 11.17 | 524.3476 | C_28_ H_48_ D_4_ O_3_ Si_3_ | G_III |
| #29 | 11.45 | 434.2969 | C_25_ H_38_ D_4_ O_2_ Si_2_ | G_III |
| #30 (MENT) | 11.84 | 436.3123 | C_25_ H_40_ D_4_ O_2_ Si_2_ | G_III |
| #31 | 12.44 | 524.3476 | C_28_ H_48_ D_4_ O_3_ Si_3_ | G_III |
| #32 | 13.06 | 524.3471 | C_28_ H_48_ D_4_ O_3_ Si_3_ | G_III |
| #33 | 13.56 | 524.3475 | C_28_ H_48_ D_4_ O_3_ Si_3_ | G_III |
| #34 | 13.84 | 525.3536 | C_28_ H_47_ D_5_ O_3_ Si_3_ | G_III |
| #35 | 14.11 | 229.0730 | C_9_ H_17_ O_3_ Si_2_ | G_III |
| #36 | 14.42 | 525.3534 | C_28_ H_47_ D_5_ O_3_ Si_3_ | G_III, S_III |
| #37 | 14.92 | 527.3692 | C_28_ H_49_ D_5_ O_3_ Si_3_ | G_III |
| #38 | 15.68 | 525.3536 | C_28_ H_47_ D_5_ O_3_ Si_3_ | G_III |
| #39 | 15.81 | 609.3612 | C_31_ H_57_ D_2_ O_4_ Si_4_ | G_III |
| #40 | 16.01 | 229.0730 | C_5_ H_19_ D O_4_ Si_3_ | G_III |
| #41 | 16.54 | 504.2860 | C_27_ H_42_ D_3_ O_3_ Si_3_ | G_III |
| #42 | 16.80 | 521.3241 | C_28_ H_45_ D_4_ O_3_ Si_3_ | G_III |
| #43 | 17.41 | 467.2767 | C_24_ H_39_ D_4_ O_3_ Si_3_ | G_III |
| #44 | 17.52 | 562.3289 | C_30_ H_50_ D_2_ O_4_ Si_3_ | G_III |
| #45 | 8.11 | 439.3344 | C_25_ H_41_ D_5_ O_2_ Si_2_ | G_IV |
| #46 (M2) | 9.64 | 437.3188 | C_25_ H_39_ D_5_ O_2_ Si_2_ | G_IV |
| #47 (M1) | 10.38 | 439.3345 | C_25_ H_41_ D_5_ O_2_ Si_2_ | G_IV, S_IV |
| #48 | 13.18 | 348.2767 | C_22_ H_30_ D_5_ O Si | G_IV |
| #49 | 13.98 | 474.2932 | C_27_ H_40_ D_3_ O_3_ Si_2_ | G_IV |
| #50 | 17.08 | 499.3380 | C_26_ H_45_ D_5_ O_3_ Si_3_ | G_IV, S_IV |
